# Physical Activity Programs during Pregnancy Are Effective for the Control of Gestational Diabetes Mellitus

**DOI:** 10.3390/ijerph17176151

**Published:** 2020-08-24

**Authors:** José Alberto Laredo-Aguilera, María Gallardo-Bravo, Joseba Aingerun Rabanales-Sotos, Ana Isabel Cobo-Cuenca, Juan Manuel Carmona-Torres

**Affiliations:** 1 Department of Nursing, Physiotherapy and Occupational Therapy, Facultad de Ciencias de la Salud, Universidad de Castilla-La Mancha, Av Real Fábrica de Sedas s/n, 45600 Talavera de la Reina, Spain; josealberto.laredo@uclm.es (J.A.L.-A.); mariagallardobravo1997@gmail.com (M.G.-B.); 2 Grupo de Investigación Multidisciplinar en Cuidados (IMCU), Universidad de Castilla-La Mancha, Campus de Fábrica de Armas, Av de Carlos III, nº 21, 45004 Toledo, Spain; anaisabel.cobo@uclm.es (A.I.C.-C.); juanmanuel.carmona@uclm.es (J.M.C.-T.); 3 Department of Nursing, Physiotherapy and Occupational Therapy, Facultad de Enfermería, Universidad de Castilla-La Mancha, Campus Universitario s/n, 02071 Albacete, Spain; 4 Grupo de Actividades Preventivas en el ámbito Universitario de Ciencias de la Salud (GAP-CS), Universidad de Castilla-La Mancha, Campus Universitario s/n, 02071 Albacete, Spain; 5 Department of Nursing, Physiotherapy and Occupational Therapy, Facultad de Fisioterapia y Enfermería, Universidad de Castilla-La Mancha, Campus de Fábrica de Armas, Av de Carlos III, nº 21, 45004 Toledo, Spain

**Keywords:** active pregnancy, exercise, gestational diabetes mellitus, nursing, physical activity, pregnant

## Abstract

Gestational diabetes mellitus has an incidence of 14% worldwide and nursing is responsible for its monitoring during pregnancy. Excessive weight gain during pregnancy is directly related to gestational diabetes mellitus development. Gestational diabetes mellitus (GDM) has negative repercussions on the evolution of the pregnancy and the fetus. The objective of this systematic review is to establish how physical activity influences pregnant women with gestational diabetes mellitus and to analyze what benefits physical activity has in the control of gestational diabetes mellitus. A systematic search was carried out in different databases (Cochrane, Superior Council of Scientific Investigations (CSIC), EBSCOhost, Pubmed, Scopus, Web os Science, and Proquest) for papers published within the last 12 years, taking into account different inclusion and exclusion criteria. Six randomized controlled studies and one observational case-control study of a high quality were selected. Fasting, postprandial glucose and HbcA1 were assessed, as well as the requirement and amount of insulin used. Thus, there is a positive relationship between the performance of physical activity and the control of gestational diabetes mellitus. Resistance, aerobic exercise, or a combination of both are effective for the control of glucose, HbcA1, and insulin. Due to the variability of the exercises of the analyzed studies and the variability of the shape of the different pregnant women, it does not permit the recommendation of a particular type of exercise. However, any type of physical activity of sufficient intensity and duration can have benefits for pregnant women with GDM. Pregnant women with gestational diabetes mellitus should exercise for at least 20–50 min a minimum of 2 times a week with at a least moderate intensity.

## 1. Introduction

Life expectancy has increased and people live longer, although this increase in life has increased the number of chronic diseases, each time at an earlier stage [1]. It has been observed that one of the most frequent chronic diseases, diabetes, has increased in adolescents and is estimated for a more than 4-fold increase in the next few decades [2]. Diabetes is a chronic disease suffered by 283 million people, a number that is expected to reach 592 million in 2035 [3]. This chronic disease appears when the pancreas is incapable of producing enough insulin, or when it is not used effectively by the body. Spain, together with the United States, is one of the countries with the highest fasting diabetes and glucose index [4].

The American Diabetes Association (ADA) defines gestational diabetes mellitus (GDM) as diabetes diagnosed in the second or third trimester of pregnancy that was not clearly overt diabetes prior to gestation [5]. GDM can reach up to 14% of the population worldwide [6]. Insulin may or may not be necessary in this case, regardless of the degree of metabolic disorder. In addition, this pathology can persist once the pregnancy has ended [7].

Pregnancy causes major biochemical changes that cause a decrease in insulin sensitivity, offset by an increase in insulin production. Good control of a woman with GDM through diet and exercise can avoid the use of insulin, requiring only 20–30% insulin [8]. According to this statement, the consensus of the Spanish Group on Diabetes and Pregnancy defines that GDM should be treated with dietary measures and physical exercise first. However, this does not mean that pharmacological treatment, such as insulin, is not necessary when adequate metabolic control is not achieved with the above indications [9,10]. Nurses and midwives, among other professionals, are in charge of monitoring a pregnancy and carrying out the diagnostic tests for GDM—indications for physical activity—and are in the closest contact with pregnant women [11].

It is important to treat this complication of pregnancy because patients with GDM are at an increased risk of developing type II diabetes after pregnancy [7]. There is also the possibility that the child will suffer complications as macrosomia, impaired intrauterine growth, obstetric trauma, hyperbilirubinemia, hypoglycemia, intection, and a length of stay in the intensive care unit [12]. A combination of diet and exercise reduces excessive weight gain during pregnancy and GDM because weight gain is directly related to GDM development [13,14]. In addition, obese women tend to have an unbalanced glucose tolerance and higher insulin resistance during pregnancy than those with a healthy weight [7]. Thus, pregnant women who are overweight or obese have between 2.14 and 3.56 times more risk of GDM than those with a healthy weight. If we focus on percentages, the prevalence of GDM would be 0.7% in women with a healthy weight, 2.3% for those overweight, 4.8% for those who are obese, and 5.5% for those with a body mass index higher than 35 [15].

There has always been a controversy about exercising during pregnancy [16]. For this reason, around 80% of pregnant women are physically inactive, increasing this inactivity during the last trimester of pregnancy [17]. However, today it is known that there are many benefits that exercise offers to both the fetus and the mother. Among the maternal benefits are a general decrease in cramps, lower back pain, oedema, depression, urinary incontinence, the duration of labour, and constipation as well as the number of caesarean sections of the mother [18]. Physical activity has benefits for the fetus: decreased fat mass, improved stress tolerance, and advanced neurobehavioral maturation, among others [19]. In addition, physical activity reduces the rate of GDM to those who perform it between three to twelve months regularly before or during the gestation period [20]. 

Physical exercise can be carried out safely by pregnant women preventing excessive weight gain, macrosomia, high blood pressure, GDM, respiratory distress syndrome, neonatal hypoglycemia, and hypocalcemia [21,22,23,24]. The benefits of physical activity requires physical activity for 30 min at a moderate intensity for five days, or 150 min of aerobic activity every week on average, depending on the women’s physical activity level or fitness status before pregnancy [25]. It should not be noted that both the intensity and the type of activity depend on each person and should always be recommended individually [26]. 

On the other hand, it should not be forgotten that sometimes there are severe restrictions to exercise. These circumstances include heart or lung problems, cervical incompetence, threatened labour, or premature rupture of the membrane, pre-eclampsia or severe anaemia. For this reason, it is necessary to follow the instructions of professionals, such as midwives and gynaecologists, who offer tailored safe recommendations on the sports or activities each pregnant woman can carry out [26].

This review investigates the influence of physical activity in women with GDM so in the future its importance and adherence to it are recognized. It also discusses the controversy of physical activity in pregnancy in a recent meta-analysis which indicates that exercise can only perform a preventive approach in GDM [16]. The close relationship between the nursing community and midwives has great potential to influence pregnant women and to profoundly improve their health to face the battle against excess weight in pregnancy and GDM [11]. In addition, professionals can reach an agreement on the safest and most effective recommendations, since currently there is still controversy about the screening protocols or diagnostic criteria of GDM and the best or most successful treatment for this pathology [27].

### Objectives: Peak Question

Due to the controversy over whether it is beneficial for pregnant women with GDM to carry out physical activity or not, this review aims to find out how physical activity influences pregnant women with this pathology and to analyze what benefits exercise, or physical activity, brings to the GDM control.

## 2. Materials and Methods

### 2.1. Information Sources

The following review was carried out through a bibliographic search that started in September 2019 and ended in December 2019 in the following health-related databases: Pubmed, WOS (Web Of Science), Scopus, Cochrane Library, Proquest, CSIC, EBSCOhost.

### 2.2. Search Strategy

The search strategy was based on the search string with keywords in the MeSH/DeCS descriptors of the databases mentioned earlier. The string was completed with the Boolean operators AND and OR.

Table 1 shows the used PICO criteria, where it shows how each part of the search chain belongs to each of the peak criteria, following the proper structure with its corresponding keywords.

### 2.3. Inclusion Criteria 

The inclusion criteria include publication within the last 12 years (from 1 January 2008, to 31 December 2019), the population of pregnant women without age range with diagnosed GDM in pregnancy and not a priori or after gestation, a specific type of activity or physical exercise carried out as the intervention, and the studies in English or Spanish.

The exclusion criteria are animal tests and an unspecific amount or type of physical activity by women. The articles that did not differentiate the physical activity from the diet of the pregnant women were also discarded. All GDM prevention studies and systematic reviews were excluded due to inadequate scientific quality.

### 2.4. Selection of Studies and Collection of Data

**Figure 1:**
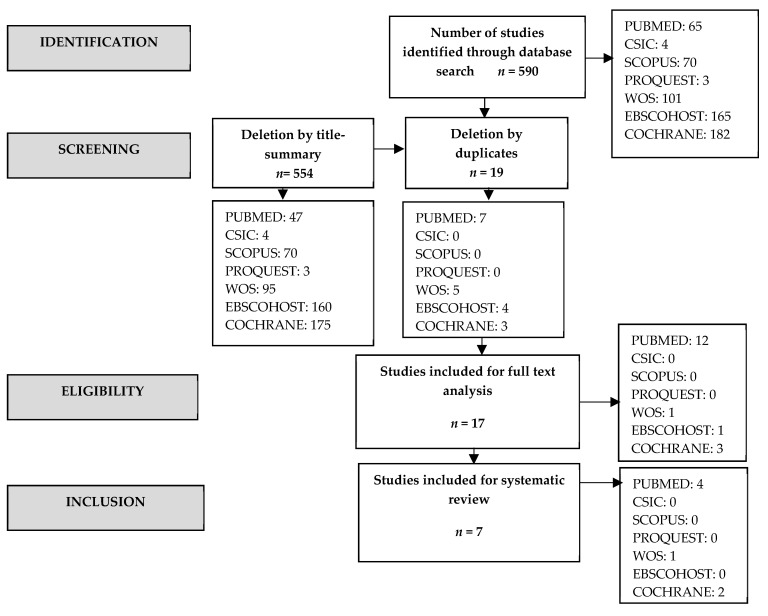
Prisma flow chart.

After an exhaustive search, a total of 590 results were obtained, of which 554 were eliminated by title and summary, leaving a total of 36 (Figure 1). Nineteen duplicates were removed. The review was conducted independently by two investigators using the inclusion and exclusion criteria of this review.

Thus, a total of 17 articles were chosen for the systematic review. However, 10 of them were discarded for not meeting the established inclusion criteria.

The results were structured under a standardized registry using author, year, type of study, objective, randomization, blind, country, duration of the study, women with GDM, weeks of gestation at study start and at end, activity or exercise, intervention, and glucose levels.

### 2.5. Assessment of the Quality of Studies: Detection of Possible Bias 

Rating scales have been made to assess the quality of the studies. The PEDRO scale (11 items) was used out for randomized controlled trials (RCTs) [28]. The selection criteria were taken into account if the study population was randomized and the study was blinded for the intervention. The groups were similar at the start of the study and all subjects, therapists or those who collected the data, and evaluators were blinded as to whether there was a high proportion of population lost during the study.

In addition to the PEDRO scale, another type of scale known as the Newcastle–Ottawa scale for case-control [29] was used since 1 study of the 7 is an observational case-control study.

These scales have been carried out by one reviewer and analyzed by a second reviewer to detect possible biases.

Low risk of bias characterizes all the included articles according to the used scales.

### 2.6. Analysis of Data and Levels of Evidence

The degree of evidence depends on factors, e.g., the type of study and the methodological quality. To assess the level of evidence, a qualitative assessment was made using the SIGN scale (Scottish Intercollegiate Guidelines Network) [30]. According to this scale, all the selected RCTs are of high evidence since, as mentioned in the previous section, all of them have a low risk of bias, as does the observational case-control study.

## 3. Results

After an exhaustive search in the different databases, a total of 590 results were obtained, of which seven were finally selected to carry out this systematic review. These articles were selected using the inclusion criteria. The studied population comes from Italy, Croatia, Brazil, Thailand, Australia, Nigeria, and the United Kingdom.

Participants of the seven articles are pregnant women with GDM diagnosed during pregnancy whose ages range from 18 to 50 years. The total sample of participants is 782 women. The gestational age of women at the time of the intervention is between week 24 and the end of pregnancy (approximately week 40).

The final choice was made using the description of the exercise, and the variables of the GDM analyzed at each intervention. For this reason, the articles where the only intervention measure for GDM was a physical activity were prioritised, although fourof them combined exercise with diet [31,32,33,34]. These four articles mentioned a suitable diet: approximately 50% of carbohydrates, 20% of proteins, and 30% of fats. The article by Bo et al. [32], unlike that of Sklempe et al. [31], includes the recommended amount of fibre per day (20–25 g/day) and indicates the prohibition of alcohol during pregnancy. Furthermore, Barros et al. [33], Davenport et al. [34], and Sklempe et al. [31] specify that the total amount of food per day is around 1800–2000 Kcal.

Table 2 shows the characteristics of the selected studies.

The duration of the interventions varies between 6 and 16 weeks; therefore, a specific duration cannot be established. Regarding exercise, there are two types of modalities different from this: aerobic activity (AA) and resistance exercise (RE) or a combination of both, as in the study by Sklempe et al. [31] Three of the seven interventions mention aerobic activity as an intervention measure in the control of GDM [34,36,37]. The aerobic activity of the study by Halse et al. [36] consists of using a stationary bicycle with intervals of greater intensity with a final duration of training 45 min in the last quarter. However, the activity of Davenport et al. [34] consists of walking 3–4 times a week for about 40 min. In another study [33], the effect of an elastic band used to perform a circuit of 8 resistance exercises is analyzed, where the duration of the training increases as the pregnancy progresses. 

Sklempe et al. combine both modalities (AA and RE) and also include pelvic and stretching exercises with corresponding relaxation [31]. This study includes the most exercises concerning the type of intervention and reflects that at the end of pregnancy there is no significant difference between groups in the fasting glucose levels but there is a significant difference in postprandial glucose levels in the EG, as shown in Table 2. However, it is necessary to take into account the limitations of the study by Sklempe et al.: the study population is small, and there is no indication of participants requiring insulin during the intervention. Even so, it presents strengths such as fasting and postprandial glucose values which are lower in the intervention group, with a greater difference in postprandial glucose. It should be noted that the intensity of exercise was moderate.

The type of study activity done in Thailand is yoga [35]. This form of exercise has been included within the resistance exercise modality. For eight weeks, the studied population performed this exercise twice a week for 50 min. The variables analyzed are fasting, postprandial glucose, and glycated haemoglobin (HbA1c). These three variables are lower in the intervention group, with a significant difference (*p* = 0.012; *p* = 0.001; *p* = 0.038, respectively). Therefore, it can be affirmed that yoga is an effective exercise to control these variables. On the other hand, Bo et al. [32] studied four groups specified in Table 2, but only group B (GB) and group BE (GBE) exercised. Thus, when comparing the variables, we differentiate between those who exercise and those who do not. Their exercise is walking every day for 20 min, and the results of fasting, postprandial glucose, and HbAc1 are lower in the exercise groups, but also with significant differentiation (*p* < 0.001). 

Finally, the randomized controlled study of Daniel et al. [37] analyzes two randomly chosen groups, experimental and control. The experimental group performs the aerobic exercise, in this case, dance, for eight weeks. Compared to the control group who do not perform physical activity during this period the results are better for the experimental group for fasting glucose levels at the fourth and eighth week of physical activity, however, there are no differences between groups at the beginning. Thus, performing this type of exercise 40–60 min a week at a moderate intensity considerably reduces fasting glucose in pregnant women with GDM.

## 4. Discussion

This systematic review confirms the benefits of physical activity on fasting, postprandial glucose, and HbAc1 control in pregnant women with GDM during pregnancy. However, we cannot recommend a specific type of exercise, since the results of all the studies show similar benefits of physical activity during pregnancy in women with GDM. Only two studies [33,34] analyze the amount of required insulin to control the GDM. Both studies agree that the amount of insulin is less in the group that performs the physical activity. However, this requirement remains controversial since Davenport et al. [34] show that both the walking group and the one that does not walk still require insulin. At the same time, the study by De Barros et al. [33] indicates that the insulin requirement is lower in the exercise group that has a more intense physical activity. 

However, the two authors [33,34] agree that aerobic and resistance physical activity or a combination of both are beneficial to control GDM values. 

### 4.1. Physical Activity and Psychological Factors in Pregnant Women

In addition to the benefits of physical activity on GDM, other benefits of physical activity in pregnant women have been found, e.g., the psychological benefits [38,39]. In one of the studies [38] of the 50 pregnant women who exercised, 35 indicated that their perception of health was excellent, and only 5 of 51 women who did not exercise perceived their health to be very good. Furthermore, Nakamura [39] states that exercise reduces the appearance of depressive symptoms in pregnant women and helps to improve mood.

### 4.2. Physical Activity and GDM Prevention

When performing the search string, some of the articles were discarded because they analyzed the influence of physical activity on the prevention of GDM [40,41,42,43,44,45,46]. These studies deviated from the objective of this review—to analyze how physical activity influenced the control of GDM—and were discarded. However, prevention of GDM through physical exercise is a topic of importance closely related to the present review. Physical activity helps prevent GDM by improving glycemic control, insulin resistance, and pre-pregnancy weight gain [47]. A study indicates that an increase of 100 min of moderate to vigorous physical activity per week could reduce the risk of GDM by 9% [48]. However, the highest probability of preventing GDM by physical exercise is for pregnant women with morbid obesity [49].

In addition, the latest study claims that prevention depends on the intensity of physical activity [49]. This intensity must be moderate to reduce the risk of GDM and to improve glucose uptake. Still, another study confirms that activity levels among pregnant women are low for various reasons, e.g., lack of time, fear of injury, or willpower. In addition, physical exercise depends on the educational level, so having a low socioeconomic standard status contributes to being inactive during pregnancy [47].

### 4.3. Strengths and Limitations

The present review has some limitations, e.g., a sample size that does not allow us to generalize our results. The variability of the exercises for different interventions together with the variability of the shape of the different pregnant women makes it difficult to establish clear recommendations on the specific intensity and a specific type of exercise as the most appropriate one to control the GDM.

The strengths include the time frame of the investigation (the last 12 years), so the information is current. Furthermore, the results of the studies are similar, and their quality is high. Added to this is the considerable variability of countries where the studies have been carried out, which provides a more global vision.

Due to the paucity of articles in the last 12 years on how physical activity or exercise affects the control of GDM, more research should be done, taking into account a more representative population. In this regard, clinical trials with different types of exercise and diet are needed to assess the impact on glucose, insulin, and HbcA1 levels and to determine the most effective exercise in GDM. 

### 4.4. Implications for Clinical Practice

Physical activity protocols could improve the nursing and midwifery consultations as a support measure for pregnant patients. These protocols can prevent different problems associated with a sedentary lifestyle in pregnant women, e.g., excessive weight gain during pregnancy [14], GDM [7], high blood pressure, respiratory distress syndrome, and hypocalcaemia [21,22,23,24]. These protocols can also prevent fetus-related complications, e.g., macrosomia, impaired intrauterine growth, obstetric trauma, hyperbilirubinemia, hypoglycemia, intection, and length of stay in the intensive care unit [12]. Monitoring of pregnancy would improve, complications could be minimized, and the health costs associated with these complications would be reduced. The nurse and midwife consultation will receive all pregnant women to monitor the pregnancy, so implementing these programs from the beginning of the pregnancy will increase physical activity in this population group. Currently, such programs are not offered in the health system. These programs offered by the nurse or midwife consultation can be directed according to the stage of the pregnancy, while monitoring the potentially harmful effects of excessive physical activity.

## 5. Conclusions

Aerobic, resistance exercise, or a combination of both are effective in controlling glucose, HbcA1, and insulin. Due to the variability of the exercises of the analyzed studies and the variability of the shape of the different pregnant women, it does not allow recommending a particular type of exercise. However, any type of physical activity of sufficient intensity and duration can have benefits for pregnant women with DMG.

Pregnant women with GDM should exercise at least 20–50 min a minimum of two times a week. The intensity of the activity should be at least moderate.

While exercise provides the greatest benefit according to the analyzed studies, diet is also important to control glucose values, HbcA1, and the required amount of insulin.

Due to the scarcity of articles found on the subject under investigation, the influence of physical activity for the control of GDM requires further investigation by different professionals for better control of GDM.

## Figures and Tables

**Table 1:** PICO criterion.

Criterion (PICO)	Keywords
Population (P)	(pregnant) and (“gestational diabetes” or “type one diabetes” or “type i diabetes” or “type 1 diabetes” or “type two diabetes” or “type ii diabetes” or “type 2 diabetes”)
Intervention (I)	(“physical activity” or exercise or “exercise training” or “aerobic training” or “cardiorespiratory fitness” or “resistance training” or “resistance exercise” or “training intervention” or sport)
Outcome (O)	(“woman benefits” or “women benefits” or “woman effects” or “women effects”)

**Table 2:** Description of the type of study, intervention, sample, results, conclusions, and year of the selected studies.

Author—Year	Type of Study	Type of Intervention	Sample Size	Results	Conclusions	Quality
Sklempe et al., 2017 [31]	ECA Randomization method: it is based on the web in two groups (experimental and control) Place: 2 university hospitals in Zagreb (Croatia). Participants were not blinded. Laboratory personnel and doctors were blinded.	EG: exercises twice a week → 50–55′ session + 30′ walking/day. 20′ aerobic activity (static tape with individualized intensity) + 20–25 resistance exercises (6 exercises in 3 sets of 10 to 15 repetitions with weight and elastic band) + pelvic exercises and stretching + 10’ relaxation. CG: standard care + unsupervised exercise. Program duration: 6 w minimum. Nutrition therapy for women with GDM at the beginning: 1800 kcal per day: 20% protein (90 g), 30% fat (60 g) and 50% carbohydrates (225 g), distributed in three main meals and three snacks. Postprandial and fasting glucose levels were measured 1 or 2 times a month during pregnancy.	*N* = 38 (18 EG, 20 CG) Inclusion criteria: pregnant women with GDM between 20 and 40 years. Characteristics: age (EG: 32.78 ± 3.83; CG: 31.95 ± 4.91); upper limit gestational age (30 w); diabetic family history (EG: 7; CG: 8); gestational age of diagnosis (EG: 22.44 ± 6.55; CG: 20.80 ± 6.05); sedentary lifestyle (EG: 25.16 ± 13.2; CG: 24.36 ± 17.09)	365 exercise sessions. Intensity exercise between 3 and 14 in the Borg rating. 4.42% of maximum heart rate. Walking compliance > 70%. Moderate physical activity and transport levels during the 30th to 36th week of pregnancy > EG. No pharmacological treatment. Average fasting glucose < EG (*p* = 0.367). Postprandial glucose < EG (*p* < 0.001) No differences in weight gain or fat mass between both groups. No significant correlation was found between glycemic parameters and the duration of the intervention, adherence to the protocol or the number of assisted exercise sessions.	Aerobic exercises + resistance exercises → benefits for women with GDM. The EG had lower postprandial glucose levels at the end of pregnancy (*p* < 0.001). There was no significant difference between groups in the level of fasting glucose at the end of pregnancy.	10/11
Youngwanichsetha et al., 2014 [35]	ECA Randomization method: computer program and opaque envelopes. Place: tertiary hospital in southern Thailand.	CG: standard diabetes care. EG: standard diabetes care+ diet+ yoga during 8 w. 2 exercise sessions of 50’. Measures: fasting, postprandial glucose, and HbcA1.	180 (90 CG; 90 EG) Inclusion criteria: pregnant women with GDM between 24–30 w (no insulin). Pregnant women who have no more complications in their pregnancy. Characteristics: mean age 32.58 (SD = 5.01) CG; 31.24 (SD = 4.54) EG. Plasma glucose after 100g test CG = 89.18 (SD = 12.84) and EG = 89.36 (SD = 13.19).	Fasting glucose average: EG: 83.39 mg/dL (SD = 17.69). CG: 85.85 mg/dL (SD = 17.94). (*p* = 0.012) Postprandial glucose average: EG: 105.67 mg/dL (SD = 12.93). CG: 112.36 mg/dL (SD = 13.15). (*p* = 0.001) HbA1C average: EG: 5.23% (SD = 0.72). CG: 5.68% (SD = 0.68). (*p* = 0.038) EG < fasting, postprandial glucose, and HbcA1.	The exercise intervention program is effective in improving glycemic control.	10/11
Bo et al., 2014 [32]	ECA Randomization method: web (www.epiclin.it) Place: hospital Sant’Anna (Torino)	Multiple interventions: GD, GB, GBE, GE Diet: carbohydrates 48–50%; protein 18–20%; fat 30–35%; fiber 20–25 g/day, not alcohol. GD → diet. GB: diet + behavior→oral or written recommendations. GBE: diet +exercise → walk 20’/da slightly. GE2: diet +recommendations + exercise → walk 20’ vigorously every day + group b recommendations. Duration: until the end of pregnancy (approx. 16 w).	400 participants Inclusion criteria: pregnant women with GDM between 18–50 years and 24–26 w of gestation.	Weight, BMI, insulin, and dietary values (triglycerides and CPR concentration) → < in all groups. Postprandial glucose (*p* < 0.001) and HbA1c (*p* < 0.001) → < in the E/BE groups. Groups that do exercise (*n* = 101): Fasting glucose: 72.4 ± 10.3. Postprandial glucose: 106.1 ± 19.0. HbAc1: 4.6 ± 0.5. Groups that do not exercise (*n* = 99): Fasting glucose: 74.1 ± 10.7 Postprandial glucose: 117.2 ± 16.5 HbAc1: 4.9 ± 0.4. Groups that do exercise + diet + not recommendations (*n* = 101): Fasting glucose: 73.3 ± 10.1 Postprandial glucose: 113.0 ± 20.0 HbAc1: 4.8 ± 0.5 Groups that do exercise + diet + recommendations (*n* = 99): Fasting glucose: 73.2 ± 11.0 Postprandial glucose: 110.2 ± 17.1 HbAc1: 4.8 ± 0.4	Exercise → ↓ postprandial glucose, triglycerides and CRP concentrations. Exercise + recommendations→ no significance (*p* > 0.3). Group D → > maternal/neonatal complications. Exercise + diet → ↓ maternal/neonatal complications. Recommendations or recommendations + exercise→ no ↓ maternal/neonatal complications.	10/11
Halse et al., 2014 [36]	ECNA Place: King Edward Memorial Hospital, Perth, Western Australia	Analyze fasting, postprandial glucose levels, HbA1c and glucose and insulin response to an oral glucose load of 75 g. Conventional care based on glycemic control 2 h after breakfast, lunch and dinner + educator advice + dietitian + food and beverage daily in the 1st and last. GE → 3 sessions of supervised exercise (exercise bike at home: 5’ low intensity 55-65% warm-up + 20–30’ pedalling of > intensity with intervals between 15–60’ higher intensity every 2’. The intensity was individualized for each woman The duration of the session was increased up to 45’ in the last + 5–10’ of low intensity and gentle stretching) + 2 sessions without the supervision of 30’ until s 34 + conventional care. Supervision: TA, pre and post-exercise glucose and intake of the last meal before exercise. GC→ 35 ± 8, → walking (52%), exercise bike (40%), water exercise (5%) and yoga (3%) + conventional care.	40 participants (20 EG; 20 CG) Inclusion criteria: women with GDM, between 26–30 w of gestation, who had had an exploration of the normal anatomy in w 18, with a BMI < 45, non-smokers and not enrolled in any exercise program and who could perform physical activity. Characteristics: age (32 ± 3 → CG; 34 ± 5 → EG).	W 34 → glucose measurement and insulin response at 30, 60, 90, 120’ fasting according to GTT, HbcA1, physical activity level and nutritional status. EG → glucose response to exercise was 6.3 ± 08.mM pre-exercise at 4.9 ± 0.7mM post-exercise (*p* < 0.001). 62% of participants → capillary glucose was 1.0 mM pre to post. Half cc fasting blood glucose EG → more than the CG (*p* = 0.083) → No significance. CC postprandial glucose < in EG than in CG (*p* = 0.046). Glucose after breakfast < EG (*p* = 0.036); dinner (*p* = 0.054); lunch (*p* = 0.312) Post-intervention glucose not significant differences between EG and CG, insulin response CG (4.2 ± 2.3) and EG (5.0 ± 3.0) →(*p* > 0.05). HbcA1 → % > in post-intervention values in CG than in EG (*p* = 0.012) compared with pre-intervention values, without differences between groups (*p* > 0.05). Physical activity → EG > number of hours/w vs. CG. Feeding → CG > protein intake in the 1st s (*p* = 0.033) and last w (*p* = 0.009). HC > intake in EG in the last w (*p* = 0.035).	Cycling at home helps control postprandial glucose levels in women with GDM along with a proper diet. Supervision helps > adherence to exercise and change the lifestyle of these women. + research with + population and + exercise variety	8/11
De Barros et al., 2010 [33]	ECA Randomization method: web and opaque envelopes. Place: obstetric clinic, the university hospital of Sao Paulo (Brazil).	Diet: 7 servings → 35 cal/Kg per day + 300 Kcal/day in the day 2nd and 3rd quarter. CG → usual care. EG → wait for 90’ after eating and perform blood glucose. Exercise: resistance circuit of 8 exercises with an elastic band with 15 repetitions each exercise with a minimum of 30’ of rest and a maximum of 1’. 2nd quarter → 2 series of the circuit and 3rd quarter → 3 series. 3 times in w (1 under supervision). Moderate Intensity Glycemic profile every w. Insulin if > 30% glucose measurements > recommended value, hyperglycemia or baby weight > 75th percentile.	64 participants (32 CG; 32 EG) Inclusion criteria: women with GDM, non-smokers, sedentary between 18–45 years, without the disease, gestational age between 24–34 w. Characteristics: age CG → 32.4 ± 5.40; EG → 31.81 ± 4.87.	Insulin requirement: CG → 18 (56,3%) EG→ 7 (21.9%) Amount of insulin required IU/kg: CG → 0.49 ± 0.14 EG → 0.44 ± 0.11 Average glucose levels: CG → 102.89 ± 7.88 EG→ 100.30 ± 9.37	The resistenace exercise program with elastic band was effective in reducing the number of patients requiring insulin and to control glucose levels in women with GDM	11/11
Davenport et al., 2008 [34]	Case-control study Place: London.	2 groups were established: CG: conventional treatment: nutritionist advice every 2 w. Objectives: 2000 Kcal/day. 200 g CH in 3 meals and 3 drinks + glucose measurement + regular monitoring with your doctor. EG: hike (3–4 times at w increasing from 2’ to reach 40’ the last w. Duration: 6 w) + conventional treatment. Inclusion criteria: women with GDM, without pathology in pregnancy, and with follow-up from their doctor.	30 women → 10 EG and 20 CG. Characteristics: age → CG: 33.3 ± 5.3; EG: 33.48 ± 7.1. BMI before pregnancy → CG: 32.8 ± 5.9; EG: 32.92 ± 7.1.; Weight gained during pregnancy → CG: 12.7 ± 8.4. EG: 12.0 ± 9.7	Participants that require insulin: CG: 70%. EG: 70%. Glucose values at treatment: EG: < at the end of pregnancy tan the CG. CG capillary glucose 1 h after dinner increased at the end of the arm compared to the beginning. Amount of insulin: EG: 0.16 ± 0.13 U/kg → required less frequently. CG: 0.5 ± 0.37 U/kg.	Glucose concentration can be improved, as well as reducing the amount and frequency of insulin injection in women with GDM who walk.	7/9
Daniel et al., 2014 [37]	ECA Randomization method: random assignment to the GE/GC. Place: Owerri, Nigeria.	EG: dance exercises for 8 w. Warm-up (low-intensity aerobic exercises) and 5–10’ stretching + Dance: 10–20’ cardio-respiratory exercises (low-moderate intensity) such as fast walking. The duration increased from 40 to 60’ after 4 w + strengthening exercises (pelvic floor and abdominal muscle exercise)+ stretching and cooling 5–10’. CG: no exercise program. Fasting glucose level at the beginning. 4 and 8 w.	30 participants (15 EG; 15 CG) Inclusion criteria: women with > 24 w of gestation diagnosed with GDM suitable for exercise. Charactristics: age → EG 32 ± 3.42; CG 32.93 ± 4.61. Gestational age (weeks) → EG: 26.8 ± 0.94; CG 26.33 ± 0.98.	Fasting glucose: Start: EG(144.53; SD: 6.96); CG (145.07; SD: 8.19), (*p* = 0.85). S 4: EG (118.63; SD:10.73); CG (142.73; SD: 6.96) (*p* = 0.001) S 8: EG (87.67; SD: 11.84); CG (141.53; SD: 6.82) (*p* = 0.001)	Significant effect of the exercise program. Exercises 3 times per w between 40–60’ per session at moderate intensity reduces blood glucose.	8/11

RCT: randomized controlled study; w: week; GDM: gestational diabetes mellitus; EG: experimental group; CG: control group; BMI: body mass index; CRP: c-reactive protein; ECNA: non-randomized controlled study; GTT: glucose tolerance test; tto: treatment; cc: concentration; *n*: number; HbcA1: glycosylated haemoglobin; SD: standard deviation; *p*: a measure of statistical significance; g: grams; GD: group D; GB: group B; GBE: BE group; GE2: group E; <: minor; >: major; ′: minute; HC: carbohydrate.
